# The miR166d/*TaCPK7-D* Signaling Module Is a Critical Mediator of Wheat (*Triticum aestivum* L.) Tolerance to K^+^ Deficiency

**DOI:** 10.3390/ijms24097926

**Published:** 2023-04-27

**Authors:** Xiaotong Lei, Miaomiao Chen, Ke Xu, Ruoxi Sun, Sihang Zhao, Ningjing Wu, Shuhua Zhang, Xueju Yang, Kai Xiao, Yong Zhao

**Affiliations:** State Key Laboratory of North China Crop Improvement and Regulation, North China Key Laboratory for Crop Germplasm Resources of Education Ministry, Key Laboratory for Crop Germplasm Resources of Hebei, Hebei Agricultural University, Baoding 071000, China; lxt990209@163.com (X.L.); chenmiao_hebau@163.com (M.C.); xk901214@163.com (K.X.); sunrxhebei@163.com (R.S.); zshc@163.com (S.Z.); 15831207036@163.com (N.W.); shmzhshh@126.com (S.Z.); shmyxj@hebau.edu.cn (X.Y.)

**Keywords:** wheat (*Triticum aestivum* L.), microRNA166d, calcium-dependent protein kinases 7-D (*TaCPK7-D*), potassium starvation, potassium acquisition

## Abstract

It is well established that potassium (K^+^) is an essential nutrient for wheat (*Triticum aestivum* L.) growth and development. Several microRNAs (miRNAs), including miR166, are reportedly vital roles related to plant growth and stress responses. In this study, a K^+^ starvation-responsive miRNA (miR166d) was identified, which showed increased expression in the roots of wheat seedlings exposed to low-K^+^ stress. The overexpression of miR166d considerably increased the tolerance of transgenic Arabidopsis plants to K^+^ deprivation treatment. Furthermore, disrupting miR166d expression via virus-induced gene silencing (VIGS) adversely affected wheat adaptation to low-K^+^ stress. Additionally, miR166d directly targeted the calcium-dependent protein kinase 7-D gene (*TaCPK7-D*) in wheat. The *TaCPK7-D* gene expression was decreased in wheat seedling roots following K^+^ starvation treatment. Silencing *TaCPK7-D* in wheat increased K^+^ uptake under K^+^ starvation. Moreover, we observed that the miR166d/*TaCPK7-D* module could affect wheat tolerance to K^+^ starvation stress by regulating *TaAKT1* and *TaHAK1* expression. Taken together, our results indicate that miR166d is vital for K^+^ uptake and K^+^ starvation tolerance of wheat via regulation of *TaCPK7-D*.

## 1. Introduction

Potassium (K^+^), the most abundant cation in plants, modulates osmoregulation, stomatal regulation, enzyme activation, photosynthesis, phloem loading, and assimilate transport, promoting crop yield and quality [1,2]. Wheat (*Triticum aestivum* L.) is the most widely cultivated crop worldwide [3]. However, the widespread K^+^ deficiency of arable land has detrimental effects on sustainable agricultural development and threatens global food security.

MicroRNAs (miRNAs), typically 20–24 nucleotides long, function as small regulatory molecules that bind to target mRNA sequences and form an RNA-induced silencing complex (RISC). Previous studies substantiated that RISC negatively regulates gene expression, thereby coordinating plant development and plant–environment interactions [4,5]. In plants, miRNAs have vital regulatory roles, affecting developmental processes and stress responses [6,7,8,9,10,11,12]. Not surprisingly, they are also involved in plant responses to low-K^+^ stress. In this respect, a study revealed that in rice (*Oryza sativa* L.), the miR444a expression is decreased during exposures to low-K^+^ stress, which might lead to low-K^+^-induced plant responses through the regulation of target *MADS-box* genes [13]. In wheat grown under K^+^-deficient conditions, miR156, miR164, miR166, miR169, and miR390 expression levels are highly up-regulated, whereas the expression of miR778 and miR172 is inhibited [14]. Our previous study observed that miR408-overexpressing tobacco (*Nicotiana benthamiana*) plants exhibited significantly increased K^+^ uptake activity under K^+^-deficient conditions [15]. Nonetheless, the potential roles of miRNAs in crop responses to K^+^ starvation (LK) stress are yet to be determined.

It has been reported that miR166 is a highly conserved miRNA in land plants, with essential functions in various biological processes [16]. In Arabidopsis, during early embryonic development, *Argonaute10* (*AGO10*) represses miR165/166 activity in the entire adaxial domain and vasculature of the cotyledons and leaf primordia in the embryo proper, thus regulating the shoot apical meristem (SAM) development [17,18]. The overexpression of miR165/166 reportedly increases the root length by regulating meristematic activity in Arabidopsis [19]. It has been reported that transgenic Arabidopsis plants can develop severe pleiotropic developmental defects when short tandem target mimics (STTMs) are utilized to suppress miR166 [20]. A recent study on tomato (*Solanum lycopersicum*) revealed that disrupting *SlHB15A* using miR166 leads to aberrant ovules and parthenocarpic fruit sets [21]. In soybean (*Glycine max* L.), miR166 regulates plant height by targeting mRNAs encoding *HD-ZIP* III transcription factors [22]. Moreover, the overexpression of miR166 restricts root-to-shoot cadmium (Cd) translocation and confers Cd stress tolerance to transgenic rice plants [23]. Last but not least, miR166 influences the uptake and accumulation of nutrient ions (NH_4_^+^, Na^+^, SO_4_^2−^, Cl^−^, and PO_4_^3−^) by regulating the *Dof* transcription-factor-encoding gene *RDD1* in rice [24]. Nevertheless, how miR166 regulates plant genes in response to LK stress remains largely unknown.

Calcium-dependent protein kinases (CPKs), which are unique serine/threonine kinases in plants, contain a calcium-binding domain (CBD) and are responsive to Ca^2+^ [25,26]. Increasing evidence suggests that CPKs can enhance or inhibit channel activities and regulate ion transport across the plasma membrane [27,28]. For example, AtCPK3 in Arabidopsis impairs K^+^ uptake and transport by inhibiting AtAKT1 activities under low-K^+^ conditions [29]. In contrast, in Arabidopsis, activating AtCPK6 by Ca^2+^ can promote AtKAT2 activity in leaf vascular tissues, which helps sustain K^+^ homeostasis [30], whereas AtCPK13 phosphorylates and inhibits both AtKAT1 and AtKAT2, leading to stomatal closure [31]. The overexpression of *ZmCPK11* in Arabidopsis enhances the expression of Na^+^ and K^+^ transporter genes (*AtHKT1*, *AtSOS1*, and *AtNHX1*) [32]. These findings imply that *CPKs* are crucial for plant responses to low-K^+^ stress. However, how miRNAs regulate *CPKs* to alleviate the detrimental effects of LK in wheat remains undetermined.

This study corroborated that miR166d expression is strongly induced in wheat grown under LK conditions based on small RNA sequencing analysis. We also demonstrated that *TaCPK7-D* is targeted by miR166d, and elucidated the functions of wheat miR166d and its target gene related to LK tolerance. Our results indicate that the miR166d/*TaCPK7-D* regulatory module induces the expression of *TaAKT1* and *TaHAK1*, which increases K^+^ uptake by wheat plants under LK conditions.

## 2. Results

### 2.1. Expression of miR166d in Wheat Exposed to LK Stress

**Figure 1:**
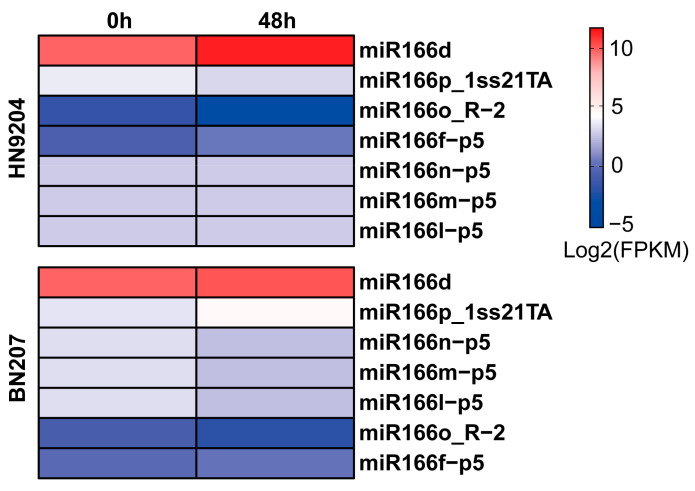
Small RNA sequencing analysis revealing that miR166 transcription is affected by LK (10 μM K^+^) stress.

To investigate the role of miRNAs in wheat tolerance to LK stress, we conducted small RNA sequencing analysis to examine the miRNA expression patterns in two wheat cultivars, HN9204 (low-K^+^-tolerant) and BN207 (low-K^+^-sensitive) [33]. Several LK-responsive miR166 members were identified (Figure 1; Table S1; Figure S1). Specifically, miR166d expression was induced 3.07-fold in HN9204 and increased little (1.29-fold) in BN207 under LK stress, implying that miR166d might participate in the response of low-K^+^-tolerant wheat plants to LK stress. Therefore, miR166d was selected as a potential key miRNA, and was functionally validated.

### 2.2. miR166d Positively Regulates Plant Tolerance to LK Stress

**Figure 2:**
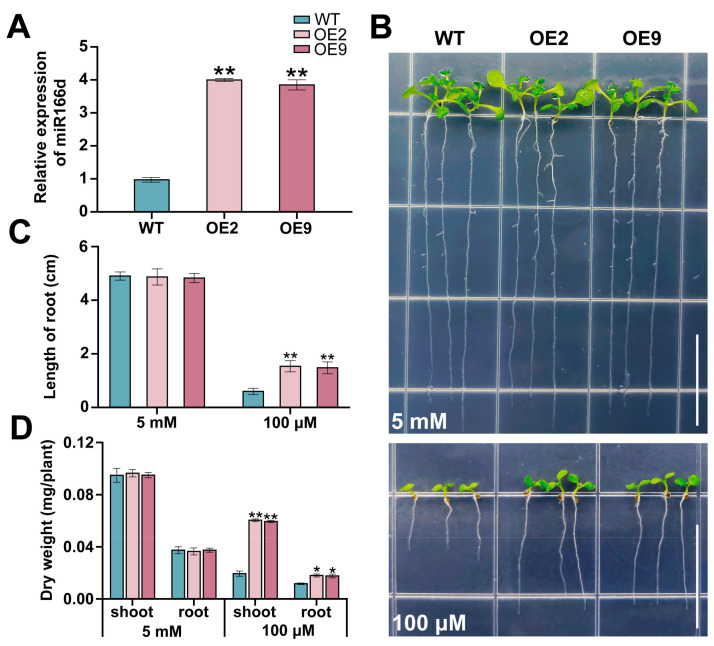
Effect of miR166d overexpression on the tolerance of Arabidopsis to K^+^ deprivation. (**A**) miR166d expression levels in WT seedlings and T3 transgenic lines (MIR166d-OE), with *AtTUB2* selected as the internal control. (**B**) Comparison of the phenotypes of the WT plants and two MIR166d-OE lines. Bars = 1.5 cm. Root lengths (**C**) and dry weights of shoots and roots (**D**) of the WT plants and MIR166d-OE lines shown in (**B**). Seedlings were grown on a medium containing 5 mM K^+^ (K^+^-sufficient conditions) or 100 μM K^+^ (K^+^ deprivation conditions) for 10 days. Data are presented as the mean ± standard deviation (*n* = 3). Student’s *t*-test was used to test the statistical significance (* *p* < 0.05, ** *p* < 0.01) between the control and treatment.

To characterize the biological function of miR166d in response to LK stress, we transformed wild-type plants (WT) with the miR166d precursor sequence (MIR166d). The expression level of the miR166d transcript was confirmed by using real-time quantitative PCR (RT-qPCR), and two lines (MIR166d-OE2 and -OE9) with a higher expression level of miR166d were used for functional analysis (Figure 2A). There were no obvious phenotypic differences between the WT and transgenic lines under K^+^-sufficient conditions. Under the K^+^ deprivation condition, however, the transgenic lines exhibited an improved growth feature compared with WT (Figure 2B). The root lengths and dry weights (shoot and root) were significantly higher in miR166d OE plants compared to the control, which is consistent with their altered growth phenotype (Figure 2C,D). Taken together, our data demonstrate that miR166d can regulate the tolerance of transgenic Arabidopsis plants to K^+^ deficiency.

**Figure 3:**
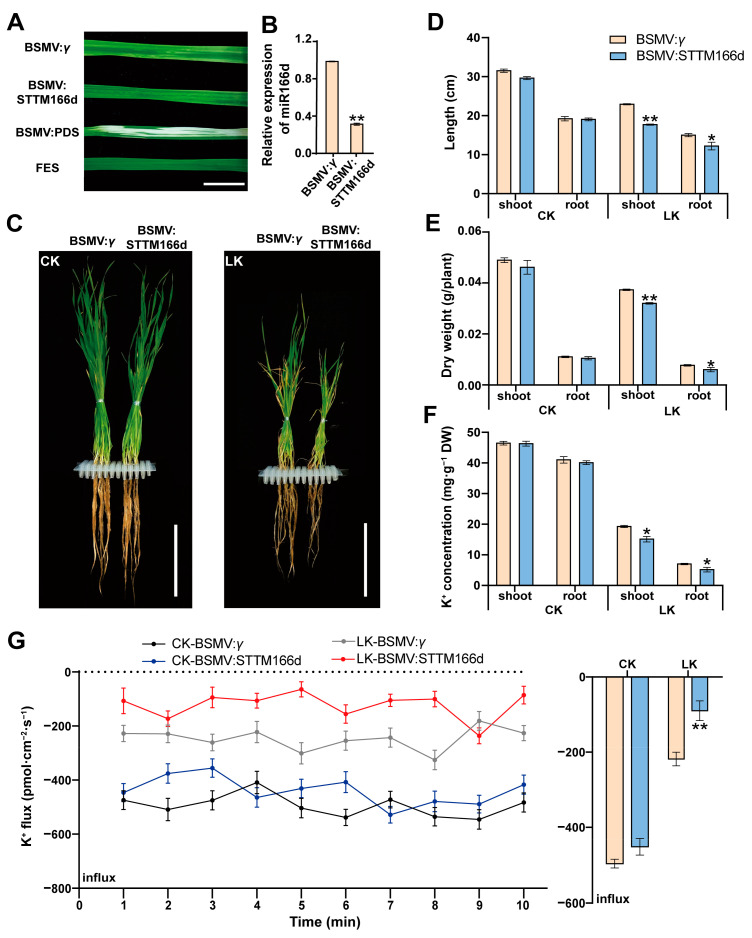
Functional analysis of wheat miR166d using the BSMV-VIGS. (**A**) Phenotypes of wheat leaves infected with BSMV:*γ*, BSMV:PDS, BSMV:STTM166d, and FES buffer at 14 days post-inoculation. Bar = 1 cm. The miR166d expression level (**B**), whole plant phenotype (**C**), lengths (**D**), dry weight (**E**), K^+^ concentration (**F**), and net K^+^ flux (**G**) of wheat seedlings infected with BSMV:*γ* and BSMV: STTM166d under CK (2 mM K^+^) or LK (10 μM K^+^) conditions at 14 days post-inoculation. Bars = 10 cm. Data are presented as the mean ± standard deviation (*n* = 3). Student’s *t*-test was used to test the statistical significance (* *p* < 0.05, ** *p* < 0.01) between the control and treatment.

We silencing miR166d in wheat plants using a *barley stripe mosaic virus* (BSMV)-based VIGS recombinant plasmid. At 14 days post-inoculation, the photobleached leaves of the plants infected with BSMV:PDS indicated that our VIGS conditions were appropriate. The leaves of plants infected with BSMV:STTM166d and BSMV:*γ* exhibited mild chlorosis (Figure 3A). Moreover, the miR166d expression level decreased by 67.78% in the BSMV:STTM166d-infected plants (Figure 3B). Compared with the control plants infected with BSMV:*γ*, the LK treatment yielded a more significant adverse effect on the morphology and growth parameters of the BSMV:STTM166d-infected plants (e.g., shoot and root lengths, dry weights, and K^+^ concentrations) (Figure 3C–F). To assess whether disrupting miR166d expression alters K^+^ uptake in wheat under LK conditions, we measured the net K^+^ flux in the roots of seedlings infected with BSMV:*γ* and BSMV:STTM166d. In response to K^+^ sufficient (CK) treatment, there was no significant difference in the net K^+^ flux between the BSMV:STTM166d- and BSMV:*γ*-infected plants during the 10 min measurement. Following LK treatment, the net influx of K^+^ in the roots was significantly lower for the BSMV:STTM166d-infected plants than for the BSMV:*γ*-infected plants during the test period. On average, the net K^+^ influx for the BSMV:STTM166d-infected plants decreased by 54.13% (Figure 3G). These findings suggest that miR166d positively regulates K^+^ uptake by wheat plants under LK stress conditions.

### 2.3. Identification of the Downstream Genes Targeted by miR166d

We predicted the miR166d targets using the online plant small RNA target analytical tool, psRNATarget (https://www.zhaolab.org/psRNATarget/, accessed on 10 February 2022). The results revealed that *TaCPK7-D* (*TraesCS2D02G207400.1*) is targeted by miR166d (Figure S2). Our degradome sequencing confirmed that miR166d could mediate the cleavage of the *TaCPK7-D* transcript (Figure S3, Table S2).

**Figure 4:**
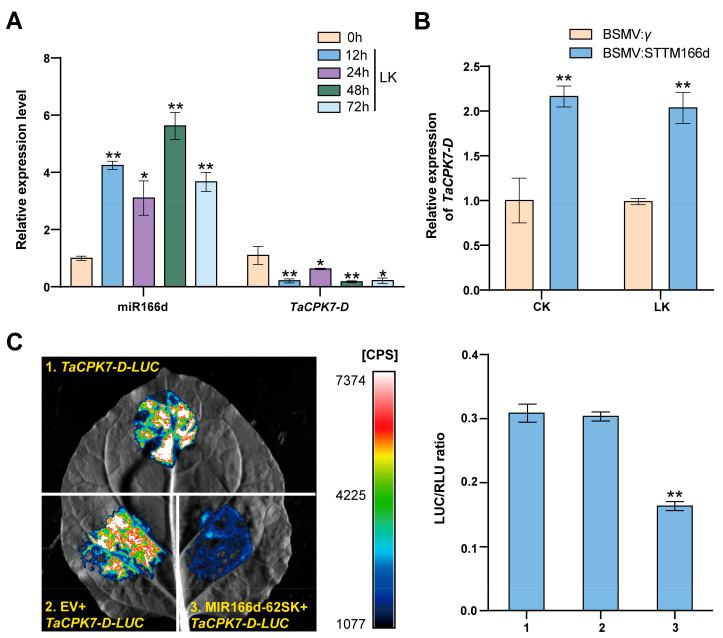
*TaCPK7-D* is directly targeted and repressed by miR166d. (**A**) Relative expression levels of miR166d and *TaCPK7-D* in wheat under LK (10 μM K^+^) conditions. (**B**) Relative expression level of *TaCPK7-D* in BSMV:STTM166d-infected plants under CK (2 mM K^+^) and LK conditions. (**C**) The miR166d/*TaCPK7-D* relationship revealed by the dual-luciferase assay; EV, *pGreenII 62-SK*; CPS, luminescence intensity; data represent the LUC/RLU ratio. Data are presented as the mean ± standard deviation (*n* = 3). Student’s t-test was used to test the statistical significance (* *p* < 0.05, ** *p* < 0.01) between the control and treatment.

RT-qPCR analysis was performed to examine the expression of miR166d and *TaCPK7-D* in response to LK treatment. In wheat roots, miR166d expression was induced substantially at 12, 24, 48, and 72 h after the LK treatment, while *TaCPK7-D* expression was decreased (Figure 4A). We also observed that the *TaCPK7-D* transcript abundance increased significantly in the BSMV:STTM166d-infected plants (Figure 4B). These findings are consistent with the prediction that *TaCPK7-D* is a target gene for miR166d in wheat. The dual-luciferase assay showed that the fluorescence intensity and luciferase activity from *TaCPK7-D-LUC* were reduced when co-transformed with MIR166d-62SK (Figure 4C). Overall, our findings indicate that *TaCPK7-D* is directly cleaved by miR166d in vivo.

### 2.4. TaCPK7-D Negatively Regulates Wheat Tolerance to LK Stress

**Figure 5:**
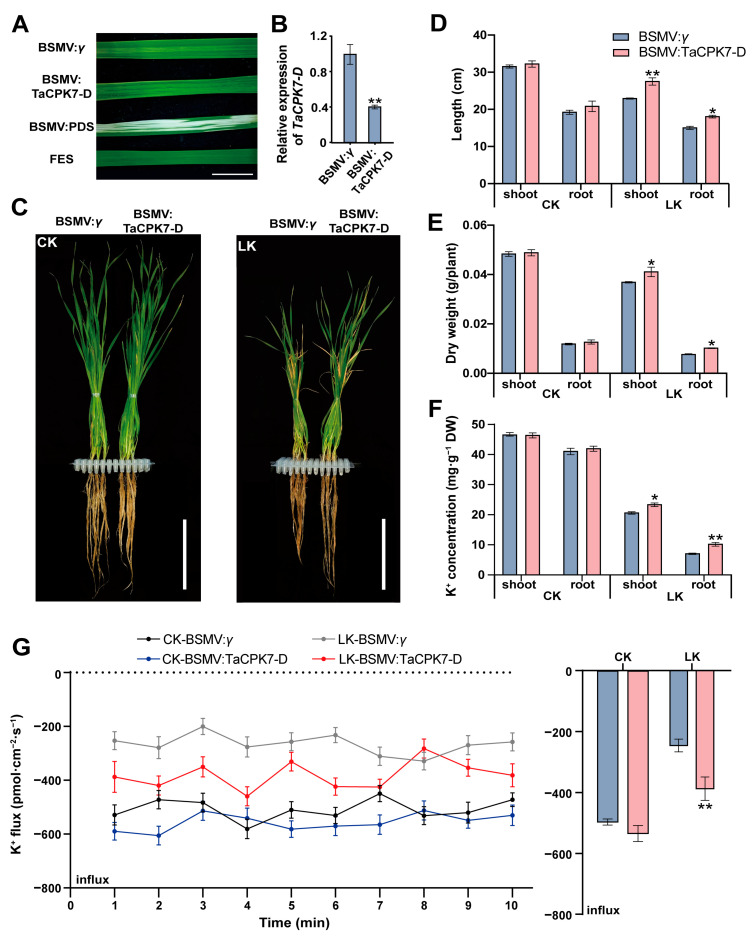
Functional analysis of wheat *TaCPK7-D* using the BSMV-VIGS. (**A**) Phenotypes of wheat leaves infected with BSMV:*γ*, BSMV:PDS, BSMV:TaCPK7-D, and FES buffer at 14 days post-inoculation. Bar = 1 cm. The TaCPK7-D expression level (**B**), whole plant phenotype (**C**), lengths (**D**), dry weight (**E**), K^+^ concentration (**F**), and net K^+^ flux (**G**) of wheat seedlings infected with BSMV:*γ* and BSMV:TaCPK7-D under CK (2 mM K^+^) or LK (10 μM K^+^) conditions at 14 days post-inoculation. Bars = 10 cm. Data are presented as the mean ± standard deviation (*n* = 3). Student’s t-test was used to test the statistical significance (* *p* < 0.05, ** *p* < 0.01) between the control and treatment.

To determine whether *TaCPK7-D* influences LK tolerance, we silenced *TaCPK7-D* using a BSMV-based VIGS recombinant plasmid. At 14 days post-inoculation, the BSMV:PDS-infected plants had photobleached leaves, indicating successful inoculation. The wheat plants infected with BSMV:TaCPK7-D had chlorosis and decreased *TaCPK7-D* expression (Figure 5A,B). Under CK conditions, the shoot and root lengths, dry weights, and K^+^ concentrations were similar between the BSMV:TaCPK7-D-infected plants and the BSMV:*γ*-infected plants. In contrast, after the LK treatment, the BSMV:TaCPK7-D-infected plants exhibited better growth parameters than the BSMV:*γ*-infected plants (Figure 5C–F).

We further measured the net K^+^ flux of the BSMV:TaCPK7-D- and BSMV:*γ*-infected seedling roots. Following CK treatment, there was no significant difference in the net K^+^ influx between the BSMV:TaCPK7-D-infected seedlings and the BSMV:*γ*-infected seedlings. However, in response to LK stress, the net K^+^ influx in the roots was significantly higher for the BSMV:TaCPK7-D-infected plants than for the BSMV:*γ*-infected plants throughout the test period. On average, the silencing of *TaCPK7-D* increased the net K^+^ influx approximately 1.54-fold (Figure 5G). Collectively, the above findings suggest that *TaCPK7-D* can negatively regulate K^+^ uptake in wheat plants subjected to LK stress.

### 2.5. The miR166d/TaCPK7-D Module Affects TaAKT1 and TaHAK1 Expression in Response to LK Stress

**Figure 6:**
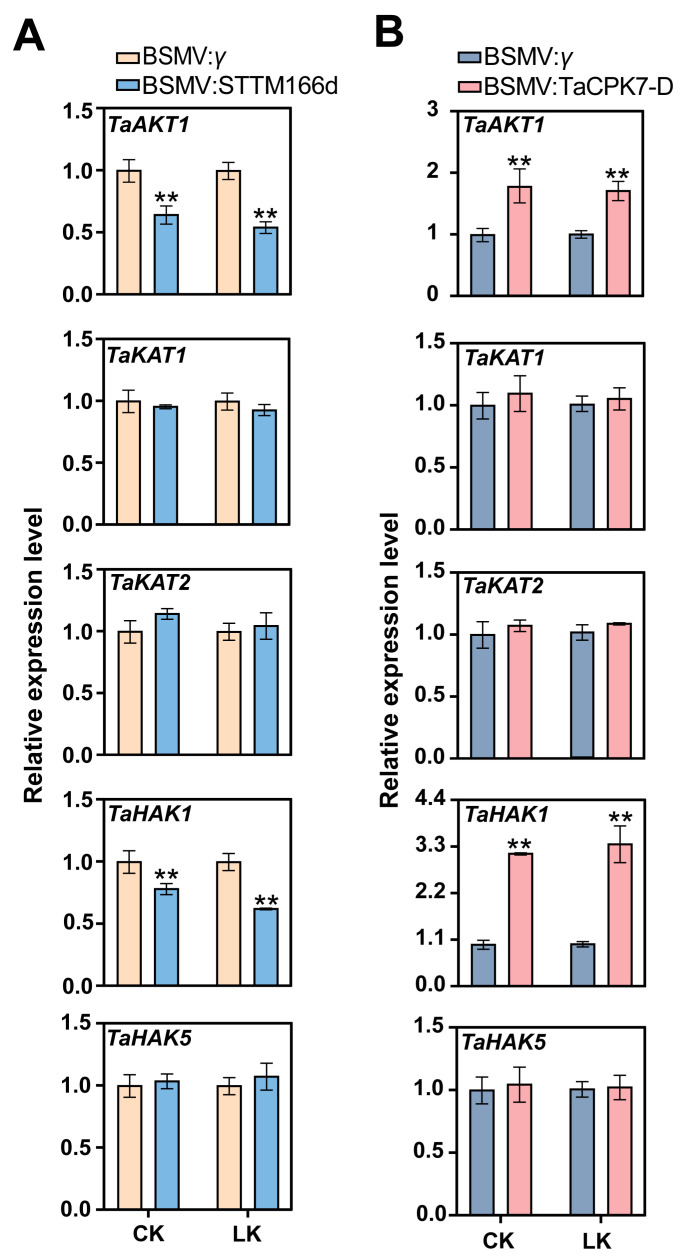
Effect of silencing miR166d (**A**) or *TaCPK7-D* (**B**) on the transcription of *TaAKT1*, *TaKAT1*, *TaKAT2*, *TaHAK1*, and *TaHAK5* in wheat exposed to LK (10 μM K^+^) stress. The transcript levels of these genes in miR166d- or *TaCPK7*-D-silenced wheat seedlings are compared with those in the control plants (BSMV:*γ*). The mRNA levels are expressed relative to the *TaGAPDH* transcript level. Data are presented as the mean ± standard deviation (*n* = 3). Student’s t-test was used to test the statistical significance (** *p* < 0.01) between the control and treatment.

To further clarify the role of the miR166d/*TaCPK7-D* module in plant responses to LK stress, we compared the expression levels of Shaker K^+^ channel genes (*TaAKT1*, *TaKAT1*, and *TaKAT2*) in the BSMV:*γ*-, BSMV:STTM166d-, and BSMV:TaCPK7-D-infected plants subjected to LK stress (Figure 6). The *TaAKT1* transcript levels were significantly lower in the miR166d-silenced plants than in the BSMV:*γ*-infected plants under CK or LK conditions, whereas they were significantly higher in the BSMV:TaCPK7-D-infected plants than in the BSMV:*γ*-infected plants. In contrast, there were no significant differences in the *TaKAT1* and *TaKAT2* transcript levels among the BSMV:*γ*-, BSMV:STTM166d-, and BSMV:TaCPK7-D-infected plants.

We further analyzed the transcription of two K^+^ transport genes (*TaHAK1* and *TaHAK5*) in BSMV:*γ*-, BSMV:STTM166d-, and BSMV:TaCPK7-D-infected plants (Figure 6). RT-qPCR indicated that compared with the BSMV:*γ*-infected plants, *TaHAK1* was expressed at lower levels in the BSMV:STTM166d-infected plants under CK or LK conditions. However, *TaHAK1* expression increased under CK conditions when *TaCPK7-D* was silenced. There was a substantial increase in the *TaHAK1* expression level in response to the LK treatment. Furthermore, *TaHAK5* expression did not differ significantly among the BSMV:*γ*-, BSMV:STTM166d-, and BSMV:TaCPK7-D-infected plants. These results imply that the miR166d/TaCPK7-D module influences K^+^ uptake in wheat by affecting the expression of *TaAKT1* and *TaHAK1* under LK conditions.

## 3. Discussion

It has long been established that K^+^ is a key nutrient for wheat growth and development. Alongside that, K^+^ deficiency worldwide has resulted in suboptimal global wheat production [34]. Over the past few years, many K^+^ deficiency-responsive miRNAs have been identified in Triticeae crops. An earlier deep-sequencing analysis of two barley (*Hordeum vulgare* L.) genotypes that differ in terms of low-K^+^ tolerance identified 207 miRNAs, of which 12 were differentially expressed between the examined barley genotypes [35]. A previous study showed that K^+^ deficiency could significantly induce miR397 and miR1118 expression, but yielded the opposite effect on miR408 and miR9778 expression [15]. Although many K^+^ deficiency-responsive miRNAs have been identified, functional analyses are needed to determine their contributions to wheat low-K^+^ stress tolerance. By degrading mRNA targets, miRNAs in plant species help regulate several biological processes [36]. The present study identified a novel regulatory module, miR166d/*TaCPK7-D*, in wheat. This module was shown to increase the expression of *TaAKT1* and *TaHAK1*, promoting K^+^ uptake under LK conditions.

An increasing body of evidence suggests that miR166 participates in multiple developmental processes or stress responses in plants [37,38]. To date, relatively few reports have described the mechanisms by which miR166 mediates plant responses to LK stress. The study identified miR166d in wheat and demonstrated its upregulation under LK conditions, with higher expression observed in HN9204 than in BN207. Thus, we speculated that miR166d plays a role during wheat responses to LK stress. Compared with the WT plants, the transgenic Arabidopsis plants overexpressing miR166d exhibited better growth under K^+^ deprivation conditions. MiR166d expression was suppressed in wheat using BSMV-VIGS. Overall, these results demonstrated that the BSMV:STTM166d-infected plants experienced more significant shoot and root growth inhibition under LK stress than the BSMV:*γ*-infected seedlings. Moreover, the BSMV:STTM166d-infected plants had a significantly lower K^+^ concentration. In addition, by comparing the net K^+^ influx in the roots, we provide direct evidence that silencing miR166d could adversely affect K^+^ uptake. Our data demonstrate that the increased expression of miR166d during exposure to LK stress may be critical for enhancing the tolerance of wheat plants to K^+^ deficiency.

The biological functions of miRNAs are mediated through their regulatory effects on specific targets. It is widely thought that miR166 modulates the genes encoding *HD-ZIP* III and *Dof* transcription factors in plants [24,39,40]. In the present study, using the psRNATarget online tool and degradome sequencing, we confirmed that *TaCPK7-D* transcripts were cleaved by miR166d. We also observed that the LK treatment exerted opposite effects on miR166d and *TaCPK7-D* expression in wheat roots. Additionally, the accumulation of *TaCPK7-D* transcripts was significantly suppressed by the expression of miR166d according to the dual-luciferase assay. Thus, our findings indicate that miR166d can cleave *TaCPK7-D* transcripts in wheat (i.e., *TaCPK7-D* is targeted by miR166d).

Calcium-dependent protein kinases (CPKs) are crucial sensors of changes to Ca^2+^ concentrations in plant cells caused by diverse endogenous and environmental stimuli. A study previously identified 20 *TaCPK* genes in bread wheat necessary for plant responses to various biotic and abiotic stresses [41]. In this respect, it has been reported that *TaCPK7*-*D* positively regulates wheat resistance to sharp eyespot infections by modulating the expression of defense-related genes [42]. Although some *CPKs* have been implicated in regulating stress responses, the functions of CPKs under LK stress conditions and the upstream regulatory factors remain unknown. In this study, we silenced *TaCPK7-D* using BSMV-VIGS and found that compared with the BSMV:*γ*-infected seedlings, the BSMV:TaCPK7-D-infected plants exhibited a significantly greater shoot and root growth and had a higher K^+^ concentration following LK treatment. Furthermore, we provided compelling evidence of increased K^+^ uptake in wheat by comparing the net K^+^ influx in the roots following the disruption of *TaCPK7-D* expression.

K^+^ channels and transporters are critical for the uptake of K^+^ in wheat. For example, *TaAKT1* contributes to the constitutive and inducible K^+^ uptake by wheat roots [43], while *TaHAK1-4A* is important for the uptake of K^+^ by LK-stressed wheat roots [44]. Another recent study indicated that *TaHAK5* promotes wheat root elongation and K^+^ uptake [45]. In addition, earlier research showed that Shaker K^+^ channels (AtAKT1, AtAKT6, AtKAT1, and AtKAT2) are regulated by CPKs [29,30,31,32,46]. We sought to explore why the K^+^ uptake of BSMV:STTM166d- and BSMV:TaCPK7-D-infected plants differed from BSMV:*γ*-infected seedlings by analyzing the expression of three K^+^ channel-encoding genes (*TaAKT1*, *TaKAT1*, and *TaKAT2*) and two K^+^ transporter-encoding genes (*TaHAK1* and *TaHAK5*) in the BSMV:*γ*-, BSMV:STTM166d-, and BSMV:TaCPK7-D-infected plants under CK and LK conditions. Compared with the BSMV:*γ*-infected plants, *TaAKT1* and *TaHAK1* were expressed at lower levels in the BSMV:STTM166d-infected plants. In contrast, silencing *TaCPK7-D* in wheat upregulated the expression of *TaAKT1* and *TaHAK1*. The downregulation of miR166d and *TaCPK7-D* resulted in changes in K^+^ concentration and a net influx in wheat, which aligns with the roles of *TaAKT1* and *TaHAK1*.

In conclusion, we developed a working model for the role of the miR166d/*TaCPK7-D* module during wheat responses to K^+^ deficiency. Compared with control plants, the plant overexpression of miR166d or silencing of *TaCPK7-D* exhibited drastically improved phenotypes, biomass and tolerance to K^+^ deficiency. In contrast, silencing miR166d expression decreased wheat plant growth and K^+^ uptake. Under K^+^ starvation conditions, miR166d expression is increased, and miR166d could effectively target and cleave *TaCPK7-D*, which resulted in decreased mRNA levels of *TaCPK7-D*. Additionally, the decrease in *TaCPK7-D* could release *TaAKT1* and *TaHAK1*, thereby enhancing K^+^ acquisition and K^+^ deficiency tolerance in wheat.

## 4. Materials and Methods

### 4.1. Plant Materials and Growth Conditions

The low-K^+^-tolerant wheat cultivar, HN9204, was selected for this study [47]. Seeds were sterilized in 75% alcohol for 5 min, rinsed twice with distilled water, and then soaked in deionized water to induce germination. After 1 week, uniformly grown plants were transferred to plastic containers for the subsequent cultivation under following hydroponic growth conditions: 20 °C, 70% relative humidity, and a 12 h light/12 h night photoperiod. Wheat materials were cultured in a modified Hoagland solution with 2 mM K^+^ (CK) or 10 μM K^+^ (LK) [48].

To analyze the miR166d and *TaCPK7-D* expression patterns, 21-day-old seedlings were treated with LK stress and root tissues were obtained at 0, 12, 24, 48, and 72 h. The samples were immediately frozen in liquid nitrogen and stored at −80 °C. For the BSMV-based VIGS assays, 7-day-old seedlings cultured under CK conditions were infected with BSMV. The infected seedlings were transferred to CK or LK conditions for 14 days. To determine the expression of miR166d, *TaCPK7-D*, K^+^ channel- and transporter-related genes, the shoot and root tissues of infected seedlings (21-day-old) were harvested for RT-qPCR analysis. Each treatment was completed using three biological replicates, and the nutrient solution was refreshed every 3 days.

Arabidopsis (Col-0) was used in this study. Seeds were sterilized in 75% alcohol and placed in a half-strength MS (1/2 MS) medium. The seeds were incubated for 2 days at 4 °C before germinating at 23 °C. After 7 days, the seedlings were transferred to pots containing vermiculite and incubated in growth chambers at 23 °C with a 16 h light/8 h dark photoperiod and a relative humidity of 70%. For the K^+^ deprivation assay, WT and transgenic Arabidopsis plants were grown for 10 days in a medium containing 5 mM K^+^ (K^+^ sufficient conditions) or 100 μM K^+^ (K^+^ deprivation conditions). The final K^+^ concentration in the K^+^ deprivation condition medium for each experiment was measured and confirmed to be 100 μM by using atomic absorption spectrophotometry.

Tobacco was used for the dual-luciferase assay. Seeds were sterilized in 75% alcohol and germinated in a 1/2 MS medium. After 7 days, the seedlings were transferred to pots containing vermiculite and grown at 25 °C with a 16 h light/8 h dark photoperiod and a 70% relative humidity.

### 4.2. Vector Construction, Plant Transformation, and Analysis of Arabidopsis Growth

We constructed vectors using the ClonExpress II One Step Cloning Kit (Vazyme, Nanjing, China). The *pCAMBIA1302* vector was modified by introducing the miR166d precursor (MIR166d) into the *Spe*I site. This modification allowed for the subsequent expression of MIR166d under the control of the 35S promoter. The recombinant plasmid was introduced into *Agrobacterium tumefaciens* GV3101 to transform Arabidopsis, using the floral dip method [49]. Transgenic plants were selected on a 1/2 MS medium supplemented with 50 µg/mL hygromycin B. For the phenotype assay, the root lengths and the shoot and root dry weights of WT and transgenic miR166d Arabidopsis plants were examined.

### 4.3. VIGS Assay of mir166d and TaCPK7-D

To silence miR166d and *TaCPK7-D*, STTM166d (96 bp) and *TaCPK7-D* fragments (233 bp) containing the *Not*I site were cloned and inserted into the BSMV:*γ* vector to generate the BSMV:STTM166d and BSMV:TaCPK7-D recombinant plasmids, respectively, as previously described [50,51]. The plasmids of BSMV:*α*, BSMV:*β*, BSMV:*γ*, BSMV:PDS and BSMV-*γ* derivatives (BSMV:STTM166d or BSMV:TaCPK7-D) were linearized with *Mlu*I and *Spe*I, respectively. A mixture comprising 1×FES buffer and BSMV transcripts was inoculated on the first fully expanded leaf of 7-day-old wheat seedlings. As a positive control, wheat seedlings were inoculated with BSMV:PDS for a time-course analysis of VIGS. The negative control wheat seedlings were infected with BSMV:*γ*. The shoot and root lengths, dry weights, and K^+^ concentrations of plants infected with BSMV:*γ*, BSMV:STTM166d, or BSMV:TaCPK7-D were analyzed.

### 4.4. Gene Expression Analysis

We used the M5 Quickspin kit (Mei5 Biotechnology, Beijing, China) to extract the total RNA. The first-strand cDNA for the miRNA and mRNA analyses was synthesized using the miRNA 1st Strand cDNA Synthesis Kit (by stem-loop) and the HiScript^®^ II 1st Strand cDNA Synthesis Kit (Vazyme), respectively. The RT-qPCR analyses of miRNA and mRNA were performed using the miRNA Universal SYBR qPCR Master Mix (Vazyme) and the ChamQ Universal SYBR qPCR Master Mix (Vazyme), respectively. Both RT-qPCR analyses were completed using the ABI QuantStudio5 instrument (Applied Biosystems, Waltham, MA, USA). The *TaGAPDH* and *AtTUB2* genes were selected as the internal controls for wheat and Arabidopsis, respectively. The miRNA and mRNA relative abundances were calculated according to the 2^−ΔΔCt^ method [52]. Primer details are provided in Table S3.

### 4.5. Dual-Luciferase Assay

The MIR166d-62SK effector construct was prepared by inserting the MIR166d sequence into the *pGreenII 62*-*SK* vector for the subsequent expression under the control of the 35S promoter. The reporter construct (*TaCPK7-D-LUC*) was generated by incorporating the miR166d target site in *TaCPK7-D* into the *pGreenII 0800*-*miRNA* vector. *Agrobacterium* strains transformed with the indicated MIR166d-62SK, *TaCPK7-D-LUC*, *pGreenII 62-SK* vector were incubated, harvested, and resuspended in the infiltration buffer of identical concentrations (OD_600_ = 0.4). *TaCPK7-D-LUC* was mixed with MIR166d-62SK or the empty *pGreenII 62-SK* vector (control at a 1:1 (v:v) ratio) and coinfiltrated into *N. benthamiana* leaves by a needleless syringe [53]. After the infiltration, the tobacco plants were incubated at 25 °C for 48 h. Then the fluorescence intensity and luciferase activity in the leaves were measured using Tanon 5200 Multi (Tanon, Shanghai, China) and the Luciferase Reporter Gene Assay Kit (Yeasen, Shanghai, China), respectively.

### 4.6. Measurement of the Net K^+^ Flux in Wheat Plants

The net K^+^ flux in wheat plants was measured using a non-invasive micro-test technology system (NMT) [54]. Wheat roots were incubated in the testing solution to equilibrate for 10 min. The microelectrode was positioned close to the wheat roots and tested for 10 min. The K^+^ flux units are pmol·cm^−2^·s^−1^, with positive and negative values representing K^+^ efflux and influx, respectively.

### 4.7. Statistical Analysis

The data were statistically analyzed using the SPSS software v22 (IBM Inc., Armonk, NY, USA). The significance between treatments was compared using Student’ s *t*-test, with the least significant difference (LSD) assessed at the level of *p* < 0.05 or *p* < 0.01.

## Supplementary Materials

The following supporting information can be downloaded at: https://www.mdpi.com/article/10.3390/ijms24097926/s1.
