# Supplementary material for: The miR166d/TaCPK7-D Signaling Module Is a Critical Mediator of Wheat (Triticum aestivum L.) Tolerance to K+ Deficiency
Source: Int J Mol Sci. 2023 Apr 27;24(9):7926. doi: 10.3390/ijms24097926 (PMC10178733; doi:10.3390/ijms24097926)
Supplement: Supplementary file 1 [file ijms-24-07926-s001.zip › Supplementary_Figures.pdf]

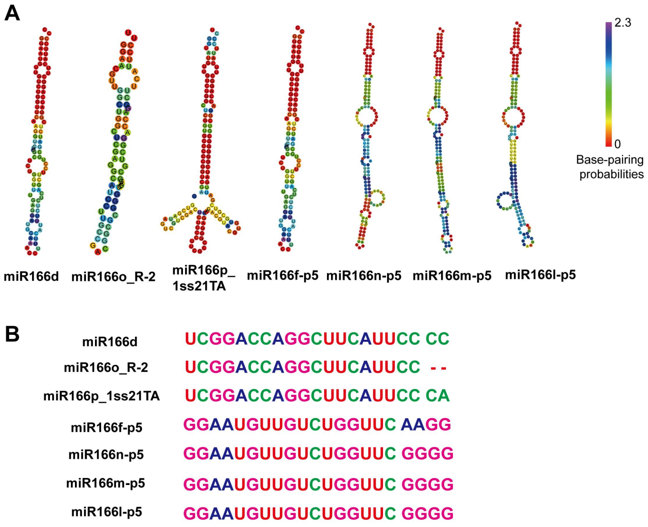

Figure S1. The secondary structure (A) and mature sequence (B) of miR166 members responsive to LK (10 μM K^+) stress in wheat roots.

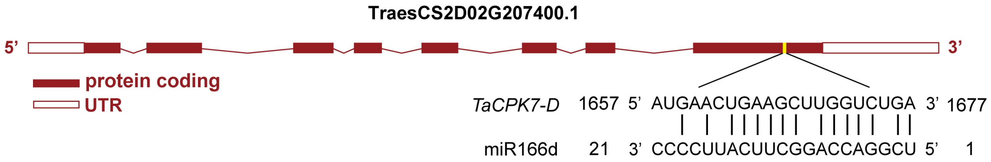

Figure S2. Interaction characterization between miR166d and *TaCPK7-D*.

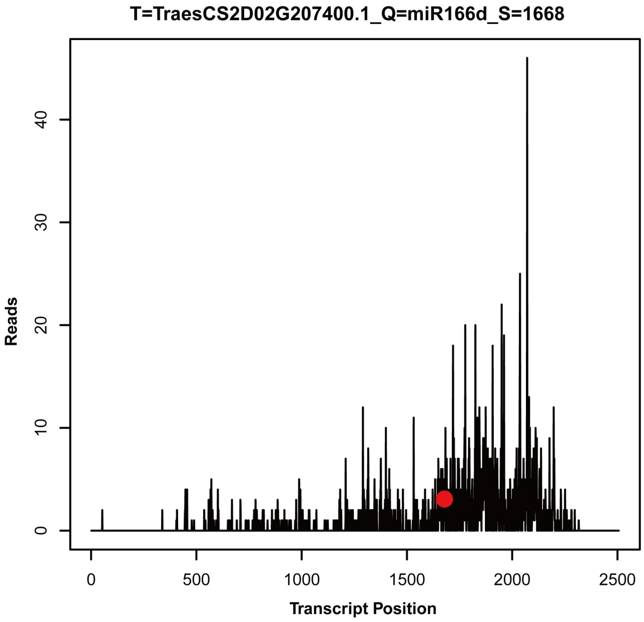

Figure S3. Degradome sequencing showing the putative cleavage site on *TaCPK7-D* transcript. The red point indicates the cleavage site.
